# Equatorial Assembly of the Cell-Division Actomyosin Ring in the Absence of Cytokinetic Spatial Cues

**DOI:** 10.1016/j.cub.2018.01.088

**Published:** 2018-03-19

**Authors:** Tzer Chyn Lim, Tomoyuki Hatano, Anton Kamnev, Mohan K. Balasubramanian, Ting Gang Chew

**Affiliations:** 1 Division of Biomedical Sciences, Warwick Medical School, University of Warwick, Gibbet Hill Road, Coventry, West Midlands CV4 7AL, UK

**Keywords:** cytokinesis, actomyosin ring, fission yeast, *S. pombe*, F-actin, swinholide-A

## Abstract

The position of the division site dictates the size and fate of daughter cells in many organisms. In animal cells, division-site placement involves overlapping mechanisms, including signaling from the central spindle microtubules, astral microtubules, and spindle poles and through polar contractions [1, 2, 3]. In fission yeast, division-site positioning requires overlapping mechanisms involving the anillin-related protein Mid1 and the tip complex (comprising the Kelch-repeat protein Tea1, the Dyrk-kinase Pom1, and the SH3-domain protein Tea4) [4, 5, 6, 7, 8, 9, 10, 11]. In addition to these factors, cell shape has also been shown to participate in the maintenance of the position of the actomyosin ring [12, 13, 14]. The first principles guiding actomyosin ring placement, however, have not been elucidated in any organism. Because actomyosin ring positioning, ring assembly, and cell morphogenesis are genetically separable in fission yeast, we have used it to derive actomyosin ring placement mechanisms from first principles. We report that, during ring assembly in the absence of cytokinetic cues (anillin-related Mid1 and tip-complex proteins), actin bundles follow the path of least curvature and assemble actomyosin rings in an equatorial position in spherical protoplasts and along the long axis in cylindrical cells and compressed protoplasts. The equatorial position of rings is abolished upon treatment of protoplasts with an actin-severing compound or by slowing down actin polymerization. We propose that the physical properties of actin filaments/bundles play key roles in actomyosin ring assembly and positioning, and that key cytokinetic molecules may modulate the length of actin filaments to promote ring assembly along the short axis.

## Highlights

• Spheroplasts lacking cytokinetic spatial cues assemble equatorial actomyosin rings
• An actin-severing compound abolishes equatorial ring assembly in spheroplasts
• Actin bundles favor the path of least curvature in the absence of cytokinetic cues

## Teaser

Lim et al. show that in fission yeast spheroplasts lacking cytokinetic spatial cues, F-actin follows the path of least curvature and assembles equatorial actomyosin rings, and that this is abolished when F-actin length is reduced chemically. Cytokinesis regulators may generate shorter F-actin for ring assembly perpendicular to the cellular long axis.

## Results and Discussion

In *S. pombe*, cell-geometry, cell-wall, and cytokinesis-positioning factors contribute to the determination of the location of the actomyosin ring [4, 12, 13, 14, 15]. To investigate where the actomyosin ring would form in the absence of all these factors (i.e., default position), we first generated spherical cells with minimal residual cell wall (spheroplasts) by enzymatically removing the cell wall in an osmotically stabilized environment. The isolated spheroplasts, which exhibited a rounded morphology, were typically cultured in medium containing 2-deoxyglucose, which is known to inhibit cell-wall assembly [16, 17, 18].

**Figure 1:**
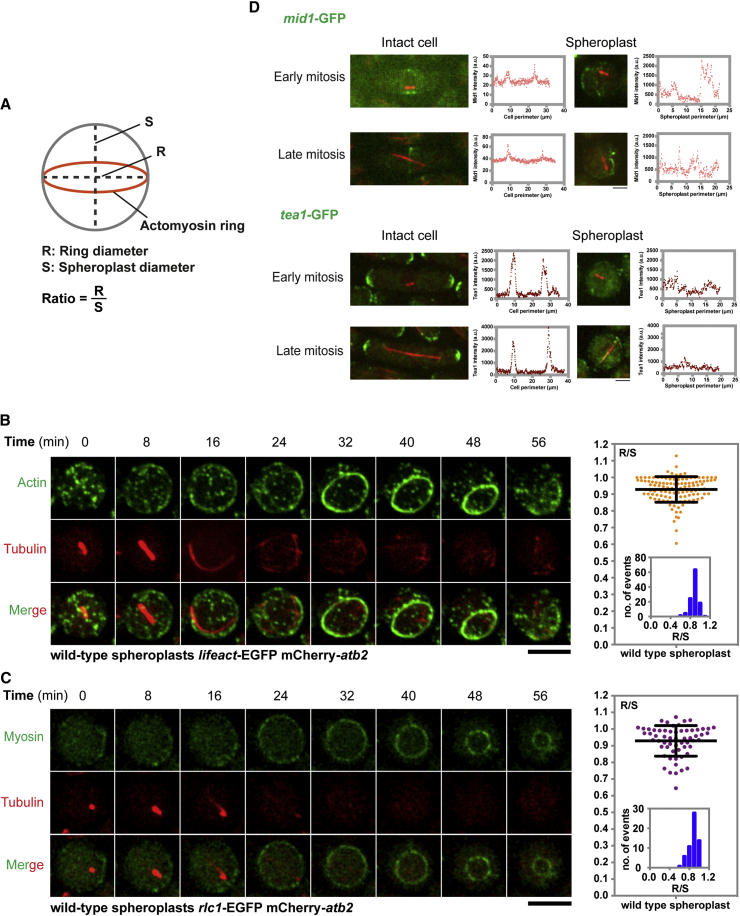
Assembly of an Actomyosin Ring at the Equator in Wild-Type Spheroplasts (A) Sketch of diameters of the rings (Rs) and spheroplasts (Ss). R and S are measured and compared. The ratio of R/S is used to indicate the size of the newly assembled rings. When the ratio is close to 1, it indicates the equatorial positioning of a ring. (B) Time-lapse microscopic images of a wild-type spheroplast expressing LifeAct-EGFP (actin) and mCherry-Atb2 (tubulin). The R/S ratio of spheroplasts is quantitated and plotted (n = 116). See also Movie S1. (C) Time-lapse microscopic images of a wild-type spheroplast expressing Rlc1-GFP (myosin) and mCherry-Atb2 (tubulin). The R/S ratio of spheroplasts is quantitated and plotted (n = 60). (D) Images of intact cells and spheroplasts expressing Mid1-GFP, Tea1-GFP, and mCherry-Atb2 (tubulin). Fluorescence intensities of Mid1-GFP and Tea1-GFP were measured along the cell perimeters, which revealed mislocalization of Mid1-GFP and Tea1-GFP in spheroplasts. Scale bars, 5 μm. Error bars indicate SD. See also Figure S1.

To study the ring assembly process, we imaged wild-type spheroplasts expressing LifeAct-EGFP (as a proxy for actin filaments) and mCherry-Atb2 (alpha-tubulin; as a marker of the cell-cycle stage) in cell suspension using time-lapse microscopy [19]. We measured and compared the ring diameters (Rs; visualized either by LifeAct-EGFP or Rlc1-GFP) to the spheroplast diameters (Ss). The diameter of the spheroplast (S) is defined as the length of a line through the center of the spheroplast that intersects two points on its circumference. The ratio of these parameters (R/S) was used to express the size of the assembled ring in relation to the diameter of the spheroplast (Figure 1A). The LifeAct-EGFP (Figure S1) and Rlc1-GFP (regulatory light chain of myosin) [20] used did not cause any overt cytokinetic phenotype in cells, and were therefore used in these studies.

When spheroplasts progressed through mitosis, as indicated by the formation and elongation of the mitotic spindle, surprisingly the actin ring assembled at an equatorial position very close to the path of maximum circumference in 90% of the spheroplasts (an R/S of 0.85 was chosen as the cutoff for equatorial localization of the ring) (R/S ∼0.93 ± 0.08; Figure 1B; Movie S1). These rings slid and disassembled eventually, as described in previous work [12, 21, 22], although these studies did not investigate ring positioning in spheroplasts. Similarly, rings containing Rlc1-GFP also assembled at the equator of spheroplasts, indicating that actomyosin rings were assembled equatorially in wild-type spheroplasts (R/S ∼0.93 ± 0.09; Figure 1C). Despite the equatorial localization of the actomyosin ring in wild-type spheroplasts, Mid1 and Tea1, key components involved in division-site placement, were barely detectable and/or were scattered throughout the spheroplast plasma membrane (Figure 1D). By contrast, Mid1 and Tea1 localized normally in intact wild-type cells (Figure 1D).

**Figure 2:**
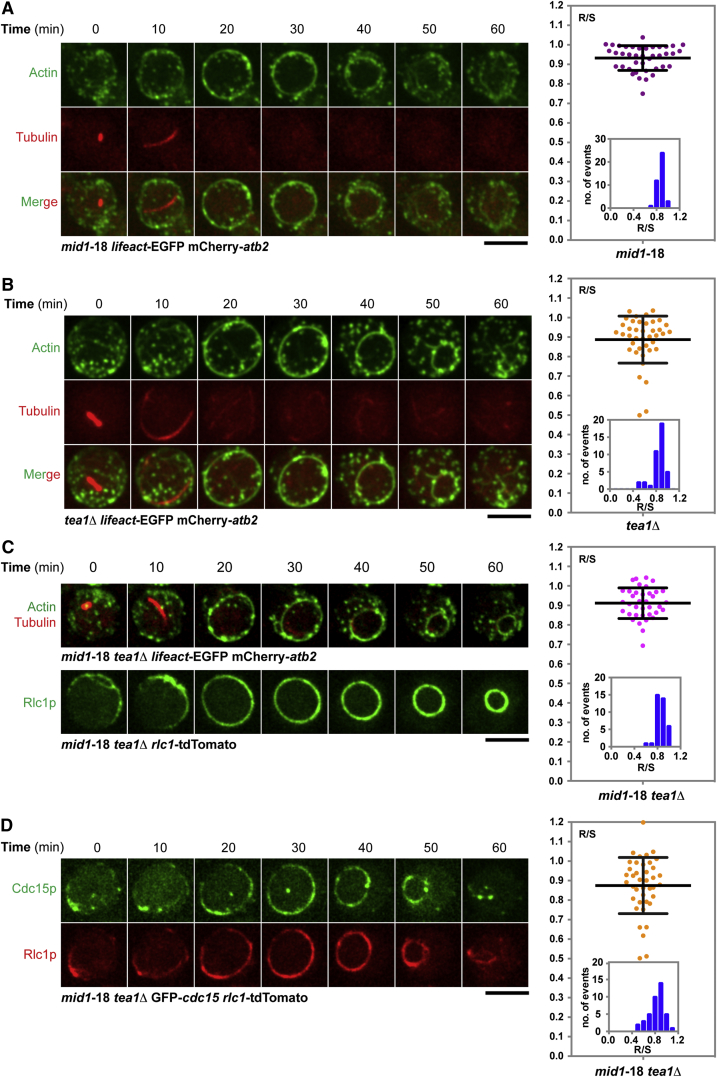
Equatorial Assembly of Actomyosin Rings in Spheroplasts Defective in *mid1* and *tea1* Functions (A) Time-lapse microscopic images of *mid1*-18 mutant spheroplasts expressing LifeAct-EGFP (actin) and mCherry-Atb2 (tubulin). The R/S ratio of spheroplasts is quantitated and plotted (n = 40). (B) Time-lapse microscopic images of *tea1*-deletion mutant spheroplasts expressing LifeAct-EGFP (actin) and mCherry-Atb2 (tubulin). The R/S ratio of spheroplasts is quantitated and plotted (n = 40). (C) Dynamics of F-actin (top) and Rlc1 (bottom) in *mid1*-18 *tea1*Δ spheroplasts. The R/S ratio of spheroplasts is quantitated and plotted (n = 37). See also Movie S2. (D) Localization of Cdc15 (top) and Rlc1 (bottom) in *mid1*-18 *tea1*Δ spheroplasts. The R/S ratio of spheroplasts is quantitated and plotted (n = 44). Scale bars, 5 μm. Error bars indicate SD. See also Figure S2.

Although Mid1 and Tea1 were either mislocalized or undetectable in wild-type spheroplasts, it was possible that slight enrichments that were not immediately apparent were causing equatorial assembly of actomyosin rings. We therefore first studied ring positioning in *mid1*-18 spheroplasts that were defective in Mid1 function. Interestingly, in *mid1*-18 spheroplasts at the restrictive temperature, the majority of the spheroplasts still assembled a ring at the equator, as in the wild-type spheroplasts. Roughly 90% of cells assembled equatorial actomyosin rings (R/S ∼0.93 ± 0.06; Figure 2A). We then tested the role of Tea1 in equatorial ring assembly and found that it also did not play a role in equatorial ring assembly (R/S ∼0.89 ± 0.12; Figure 2B). Finally, we investigated ring positioning in spheroplasts lacking both Mid1 and Tea1, and surprisingly found that ∼84% of these spheroplasts assembled equatorial actomyosin rings containing F-actin and Rlc1 (R/S ∼0.91 ± 0.08; Figure 2C; Movie S2). Interestingly, the spheroplasts lacking Mid1 or Tea1 or both assembled rings at the later stage of mitosis, which is approximately 25–30 min after mitotic entry, as indicated by the formation of short mitotic spindles (Figure S2A). There appeared to be no relationships between the axes of anaphase spindles and actomyosin rings, as we could observe a wide range of inclination angles between these two axes ranging from being parallel to being perpendicular to each other (Figure S2B; n = 20 spheroplasts). The F-BAR protein Cdc15 was also detected in equatorial rings in *mid1*-18 *tea1*Δ spheroplasts (R/S 0.878 ± 0.14; Figure 2D). Given the equatorial localization of actomyosin rings in cells defective for *mid1* and *tea1*, we tested the localization of the upstream regulators SAD-kinase Cdr2 and DYRK family kinase Pom1 in spheroplasts defective for *mid1* and *tea1*. The Cdr2 kinase and Pom1 kinase were distributed throughout the cortex or were undetectable, but were not concentrated in any equatorial pattern (Figures S2C and S2D). Thus, equatorial assembly of actomyosin rings in spheroplasts is independent of Mid1, Cdr2, Pom1, and Tea1 functions.

Given that the two major actomyosin ring-positioning mechanisms were not involved in equatorial positioning of the cytokinetic actomyosin ring in spheroplasts, we sought to determine the mechanism that positioned the actomyosin ring at the equator in spheroplasts. Recent work by Ishiwata and colleagues has shown that F-actin-containing rings can assemble at equatorial positions in cell-sized water-in-oil droplets [23]. They further proposed that actin filaments, which behave as a flexible polymer, assemble along the path of least curvature to minimize the elastic energy of actin filaments. Furthermore, these authors showed that pressing the water-in-oil droplets led to the formation of elongated actin rings that were formed perpendicular to the plane of compression, thereby again following the path of least curvature [23].

**Figure 3:**
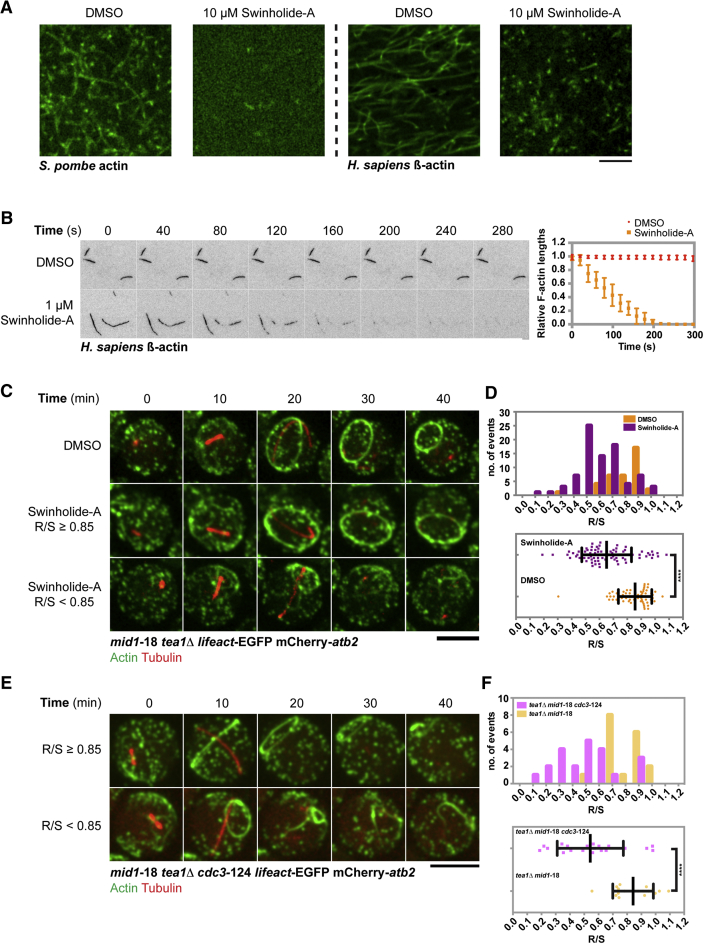
Treatment of *mid1*-18 *tea1*Δ Spheroplasts with an Actin-Disrupting Compound, Swinholide-A, Affects Equatorial Actomyosin Ring Assembly (A) Purified *S. pombe* globular actin and *H. sapiens* beta-globular actin were incubated with DMSO or with 10 μM swinholide-A in the actin polymerization buffer and imaged by fluorescence microscopy. (B) Pre-assembled actin filaments were treated with DMSO or with 1 μM swinholide-A, and imaged by total internal reflection fluorescence (TIRF) microscopy. The lengths of actin filaments after treatments were measured and normalized to their initial lengths, and their relative values were plotted as a function of time (n = 6 actin bundles/filaments for each treatment). See also Movie S3. (C) The *mid1*-18 *tea1*Δ spheroplasts expressing LifeAct-EGFP (actin) and mCherry-Atb2 (tubulin) were incubated with DMSO or with 10 μM swinholide-A. Top: DMSO control; middle: a ring assembled with a larger circumference; bottom: a ring assembled with a smaller circumference. See also Movie S4. (D) Top: a distribution of the R/S ratios of spheroplasts incubated with DMSO (n = 62) or with 10 μM swinholide-A (n = 83). Bottom: comparison of the R/S ratios of *mid1*-18 *tea1*Δ spheroplasts incubated with DMSO (n = 62) or with 10 μM swinholide-A (n = 83) (^∗∗∗∗^p < 0.0001). (E) The *mid1*-18 *tea1*Δ *cdc3*-124 spheroplasts expressing LifeAct-EGFP (actin) and mCherry-Atb2 (tubulin) were imaged at 33°C. Top: a ring assembled with a larger circumference; bottom: a ring assembled with a smaller circumference. (F) Top: a distribution of the R/S ratios of *mid1*-18 *tea1*Δ *cdc3*-124 spheroplasts (n = 22) and *mid1*-18 *tea1*Δ spheroplasts (n = 18). Bottom: comparison of the R/S ratios of *mid1*-18 *tea1*Δ *cdc3*-124 spheroplasts (n = 22) and *mid1*-18 *tea1*Δ spheroplasts (n = 18) (^∗∗∗∗^p < 0.0001). Scale bars, 5 μm. Error bars indicate SD.

We investigated whether a similar mechanism may be operating in fission yeast spheroplasts. If this were the case, treatment of spheroplasts with pharmacological agents that sever actin filaments should cause non-equatorial assembly of actomyosin rings, as shorter actin filaments may be capable of assembling into rings of higher curvature. Swinholide-A has been shown to be an actin filament-severing compound [24, 25]. We have recently developed a method to purify polymerization-competent *S. pombe* actin and human β-actin and used these actins to characterize the effects of swinholide-A (Figure 3A). Whereas actin filaments were readily observed when purified G-actin was mixed with DMSO, MgCl_2_, and ATP, such polymers were not observed when G-actin was mixed with swinholide-A, MgCl_2_, and ATP (Figure 3A). These observations suggested that swinholide-A either blocked polymerization of or caused severing of fission yeast and human actin. To specifically address whether swinholide-A severed actin filaments, we treated pre-assembled human actin filaments with swinholide-A. We observed severing of actin filaments when filaments in a flow chamber were treated with swinholide-A, but not when they were treated with the solvent, DMSO (Figure 3B; Movie S3). These results established that swinholide-A caused actin filament severing.

We then treated *mid1*-18 *tea1*Δ spheroplasts with swinholide-A or DMSO to assess the position of the actomyosin rings. DMSO-treated spheroplasts were similar to untreated spheroplasts and assembled equatorial actomyosin rings (R/S ∼0.86 ± 0.11; Figure 3C; Movie S4). Interestingly, upon swinholide-A treatment, we found two classes of actomyosin rings. In ∼14% of spheroplasts treated with swinholide-A, actomyosin rings assembled at equatorial locations. However, in ∼86% of swinholide-A-treated spheroplasts, actomyosin rings assembled at non-equatorial locations and the average R/S ratio was ∼0.65 ± 0.18, with a large number of rings even showing an R/S ratio of 0.5 (Figure 3D). We also found that partially compromising the actin polymerization factor Cdc3-profilin in *mid1*-18 *tea1*Δ (by growing at the semi-restrictive temperature of 33°C) led to assembly of non-equatorial actomyosin rings with higher curvature (Figure 3E). In spheroplasts from *mid1*-18 *tea1*Δ *cdc3*-124, ∼87% of rings assembled non-equatorially with an R/S of ∼0.54 ± 0.23 (Figure 3F). It is likely that partial compromise of Cdc3-profilin slows down actin polymerization, resulting in shorter actin filaments, which then are organized into actomyosin rings with high curvature. Collectively, these experiments suggested that long actin filaments assembled in spheroplasts during actomyosin ring assembly follow the path of least curvature to minimize the elastic energy, whereas shorter actin filaments generated upon swinholide-A treatment or when Cdc3-profilin is partially compromised allowed assembly of non-equatorial actomyosin rings.

**Figure 4:**
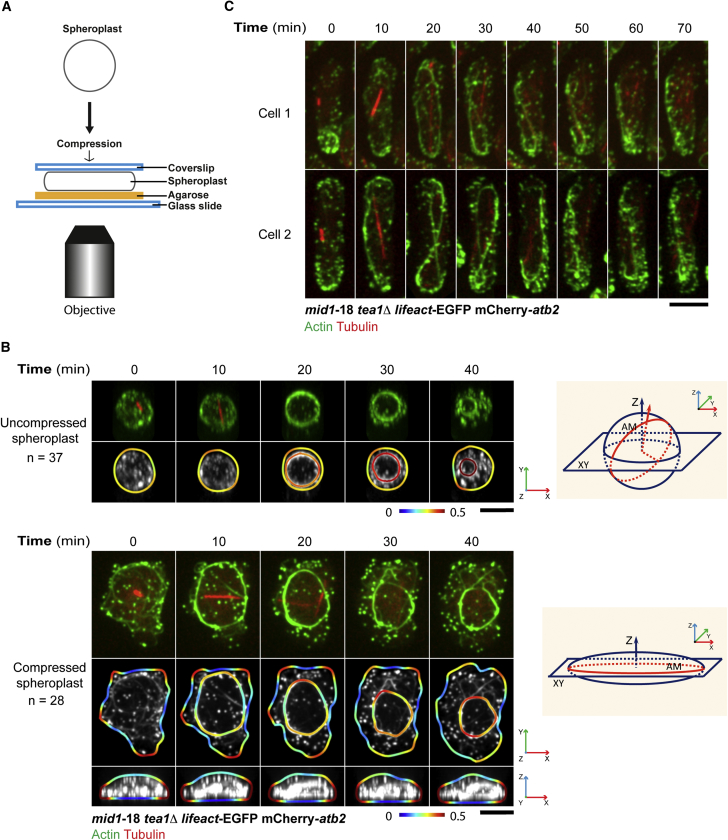
Assembly of Actomyosin Rings in Compressed *mid1*-18 *tea1*Δ Spheroplasts (A) A diagram illustrating an overview of the compression of spheroplasts by sandwiching and pressing spheroplasts between a coverslip and an agarose pad. (B) Assembly of rings at the larger cell circumference in compressed *mid1*-18 *tea1*Δ spheroplasts. The top two panels are uncompressed spheroplasts (n = 37). The bottom three panels are compressed spheroplasts (n = 28). Curvatures of the ring and spheroplast circumferences are colored coded with the rainbow look-up table. For the uncompressed spheroplasts, the image was rotated so that the ring was parallel to the imaging plane for curvature measurement. The projection axes of the displayed images are indicated by the arrows labeled with x-y-z. The two diagrams illustrate the imaging axes. AM, actomyosin ring. See also Movie S5. (C) Actin dynamics in *mid1*-18 *tea1*Δ cells expressing LifeAct-EGFP and mCherry-Atb2. Two examples are shown. Scale bars, 5 μm.

Our experiments have led to the suggestion that actomyosin rings follow a path of minimal curvature during assembly in spheroplasts lacking Mid1 and Tea1. If this were the case, compression of spheroplasts would be expected to cause ring assembly along the paths of least curvature, leading to ring assembly parallel to the imaging plane and perpendicular to the axis of compression. To test this prediction, we next compressed the *mid1*-18 *tea1*Δ spheroplasts mechanically between a coverslip and an agarose pad, as shown in the schematic in Figure 4A. Uncompressed spheroplasts assembled circular actomyosin rings of uniform curvature (uncompressed spheroplast panels, Figure 4B). Interestingly, 28 out of 28 compressed spheroplasts, upon entry into mitosis, assembled elongated actomyosin rings that were parallel to the imaging plane and perpendicular to the axis of compression, with large segments showing low local curvatures (compressed spheroplast panels, Figure 4B; Movie S5). To test whether the adoption of the path of least curvature is a property of actomyosin rings in spheroplasts, we next imaged actomyosin rings in cylindrical *S. pombe mid1*-18 *tea1*Δ cells. These cells assembled obround actomyosin rings that spanned the entire length of the cell (Figure 4C). This experiment established that even in cylindrical cells, loss of actomyosin ring-positioning factors led to ring assembly along the path of least curvature. This experiment also established that the assembly of actomyosin rings parallel to the imaging plane in compressed spheroplasts was not a result of the compression itself. Rather, the assembly of elongated actomyosin rings parallel to the imaging plane is due to the altered morphology of the spheroplasts.

Positioning of the actomyosin ring is regulated by multiple mechanisms, which ensure proper spatial coordination with genome segregation [26]. In this study, we removed the currently known cellular factors (such as cell morphology, cell wall, and two molecular positioning cues) that influence actomyosin ring positioning in *S. pombe* and asked where and how the ring was positioned in this system. Interestingly, without the influence of these cellular factors, the actomyosin ring assembled in an equatorial location along a path of least curvature. Our observations are consistent with findings of actin organization in water-in-oil droplets by Ishiwata and colleagues [23]. Furthermore, these findings are complementary to our previous work in which we have shown that the increased curvature of the actomyosin ring, gained through ring contraction, promoted its disassembly [22].

Actin filaments have a persistence length of >10 μm [27]. Given that actomyosin rings in *S. pombe* cells are composed of actin filaments ∼600 nm in length [28, 29], such smaller filaments may become easily packed along the short axis in wild-type cells. It is possible that Mid1 and/or cortical nodes not only ensure medial assembly of actin filaments [9] but also play a role in ensuring that actin filaments remain short such that they can be organized along the short axis of the cell. In the *mid1* mutant cells, and in wild-type and *mid1* mutant spheroplasts, it is possible that actin filaments are longer, causing them to organize spontaneously along the equator in a path of least curvature. Consistent with this hypothesis, we have found that generation of smaller actin filaments using pharmacological means promotes non-equatorial ring assembly in spheroplasts, as shorter actin filaments may become easily packed in smaller and more curved actomyosin rings.

In summary, our work leads to the hypothesis that the default position of the cytokinetic actomyosin ring follows the path of least curvature and that Mid1 and/or cortical nodes may play a role in restricting the size of actin filaments. How Mid1 and/or the cortical nodes regulate actin filament-length homeostasis will be a fascinating question to investigate in the future.

## STAR★Methods

### Key Resources Table

**Table:** 

REAGENT or RESOURCE	SOURCE	IDENTIFIER
**Chemicals, Peptides, and Recombinant Proteins**		

Swinholide-A	Enzo Life Sciences	BML-T125-0020
Phalloidin-biotin	Invitrogen	Biotin-XX Phalloidin B-7474
Alexa Fluor 488-C5-maleimide	Thermo Fisher Scientific	A10254
Rhodamine-phalloidin	Life Technologies	R415
LongLife Zymolyase	G-Biosciences	786-914
Lysing enzyme from *Trichoderma harzianum*	Sigma	L1412
2-deoxyglucose	Sigma	D6134
Hellmanex III	Sigma	Z805939
Avidin	Sigma	A3275

**Experimental Models: Organisms/Strains**		

*clp1::ura4+ ura4-D18 leu1-32 ade6-21* h+	Laboratory collection	MBY977
*mid1*-GFP*:ura4+* mCherry-*atb2:hph*	Laboratory collection	MBY6462
P*act1*-*lifeact*-EGFP:leu1+ mCherry*-atb2:hph ura4-D18 leu1-32* h-	Laboratory collection	MBY6659
P*act1*-*lifeact-*EGFP:*leu1+* mCherry-*atb2:hph ura4*-D18 *leu1*-32 h+	Laboratory collection	MBY7114
*mid1*-18 P*act1*-*lifeact-*EGFP:*leu1+* mCherry-*atb2:hph leu1*-32	Laboratory collection	MBY7161
*mid1*-18 *tea1::ura4+ rlc1*-tdTomato-*natMX6* h+	This study	MBY10473
*mid1*-18 *tea1::ura4+* P*act1*-*lifeact-*EGFP:*leu1+* mCherry-*atb2::hph* h-	This study	MBY10921
*mid1*-18 *tea1::ura4+* mEGFP*-cdc15:kanMX rlc1*-tdTomato-*natMX6* h+	This study	MBY10989
*tea1-*GFP:*KanMX6* mCherry-*atb2:Hph* h+	This study	MBY11194
*rlc1-*GFP:*leu1+* mCherry*-atb2:Hph* h+	This study	MBY11200
*tea1::ura4+* P*act1*-*lifeact-*EGFP:*leu1+* mCherry*-atb2:Hph* h-	This study	MBY11255
*clp1::ura4+* P*act1*-*lifeact-*EGFP:*leu1+* mCherry*-atb2:Hph* h-	This study	MBY11627
*tea1::ura4+ mid1*-18 *pom1-*GFP*:KanMX6* mCherry-*atb2:hph* h+	This study	MBY11646
*tea1::ura4+ mid1-*18 *cdc3-*124 P*act1*-*lifeact-*EGFP:*leu1+* mCherry*-atb2:Hph* h-	This study	MBY11658
*tea1::ura4+ mid1*-18 *cdr2*-GFP*:ura4+* mCherry-*atb2:hph* h-	This study	MBY11666

**Software and Algorithms**		

Prism 6.0	GraphPad	Version 6.0

**Other**		

μ-Slide 8-Well glass bottom dish	Ibidi	80827

### Contact for Reagent and Resource Sharing

Further information and requests for resources and reagents should be directed to and will be fulfilled by the Lead Contact, Mohan Balasubramanian (m.k.balasubramanian@warwick.ac.uk).

### Experimental Model and Subject Details

#### Yeast strains, medium, and culture conditions

*S. pombe* strains used are listed in the Key Resources Table. Strains were prepared by using standard fission yeast genetic techniques. Cells were cultured in rich medium YEA (5 g/L yeast extract, 30 g/L glucose, 225 mg/L adenine) until mid-log phase at 24°C for physiological analysis. Swinholide-A (Enzo Life Sciences; BML-T125-0020) was dissolved in DMSO and used at the final concentration of 10 μM in culture medium to perturb the actin cytoskeleton in spheroplasts.

#### Preparation of *S. pombe* spheroplasts

All cells used in this study were first cultured in YEA medium at 24°C to mid-log phase (OD_595_ = 0.2-0.5), and then were shifted to 36°C for 2 hr. About 20 mL of culture were spun down at 3,000 rpm for 1 min, and washed once with equal volume of E-buffer (50 mM sodium citrate, 100 mM sodium phosphate, [pH 6.0]). The cells were spun down and resuspended in 5 mL of E-buffer containing 1.2 M sorbitol. The cell suspension was incubated with 30 mg of lysing enzyme from Trichoderma harzianum containing mixtures of cell wall lytic enzymes (Sigma, L1412) at 36°C with shaking at 80 rpm for 90 min. This was followed by continuous incubation with 40 μL of LongLife Zymolyase containing β-1,3-glucanase (G-Biosciences, 1.5 U/μL) at 36°C with shaking at 80 rpm for 60 min. The cells after enzymatic digestion were spun down at 450 xg for 2 min, and washed once with 5 mL of E-buffer containing 0.6 M sorbitol. The spheroplasts were spun down at 450 xg for 2 min, and recovered in 10 mL YES medium (YEA medium with all amino acids and nucleotides supplements) containing 0.8 M sorbitol and 0.5% (v/v) of 1 M 2-deoxyglucose (Sigma, D6134) for 30 min at 36°C before microscopy imaging. The *mid1*-18 *tea1*Δ *cdc3*-124 cells in Figure 3E were prepared similarly except using an incubation temperature of 33°C.

### Method Details

#### SD confocal microscopy and TIRF microscopy

All imaging of spheroplasts and cells (including wild-type) were done using the Andor Revolution XD spinning disk confocal microscope. Imaging for all figures except those in Figures 3B and 3E were done at 36°C to heat-inactivate the Mid1-18 function. The spinning-disk confocal system was equipped with the Nikon ECLIPSE Ti inverted microscope, Nikon Plan Apo Lambda 100 × /1.45 NA oil immersion objective lens, a spinning-disk system (CSU-X1; Yokogawa), and an Andor iXon Ultra EMCCD camera. Images were acquired using the Andor IQ3 software at the pixel size of 69 nm/pixel except in Figure 2D, which was at the pixel size of 80 nm/pixel. To illuminate the fluorophores, Laser lines at wavelengths of 488 nm or 561 nm were used for the excitation. All images in the time-lapse spinning-disk confocal microscopy were acquired with Z-step sizes of 0.5 μm.

The Andor Revolution TIRF system was equipped with the inverted Nikon Eclipse microscope base, Nikon Apo 100x/1.49 NA Apo TIRF objective lens, a motorized single line Nikon TIRF module, a 60 mW 488 nm solid state Laser and an Andor Zyla sCMOS camera. Images were acquired using Andor IQ3 software at the pixel size of 65 nm/pixel. The TIRF microscopy was done at room temperature.

#### Sample preparation for live-cell imaging

To image the spheroplasts, 1-2 mL of suspension were concentrated to 20-100 μL by centrifugation at 450 xg for 2 min. About 10 μL of concentrated spheroplasts were loaded onto an Ibidi μ-Slide 8-Well glass bottom dish (Cat. No. 80827), and covered with mineral oil (Sigma, M5310) to prevent evaporation.

For swinholide-A treatment of spheroplasts in Figure 3C, spheroplasts after 30 min incubation in YES medium containing 0.8 M sorbitol and 2-deoxyglucose, were added with swinholide-A to a final concentration of 10 μM, and were imaged.

To image the compressed spheroplasts in Figure 4B and 1 μL of concentrated spheroplasts were sandwiched and compressed between a coverslip and a slide containing an agarose pad (YEA medium, 0.8 M sorbitol, 0.5% 2-deoxyglucose, 2% agarose). The slide was then sealed with VALAP (a mixture of Vaseline, lanolin and paraffin) prior to imaging.

#### Purification of human and yeast actin

Recombinant globular actin from human (β-actin) and fission yeast (Act1) was prepared by expression using a strategy described in Noguchi et al. [30], except that the methylotrophic yeast *Pichia pastoris* was used as a host for protein expression. After cell breakage, the lysates were processed similarly as described in Noguchi et al. [30].

#### Preparation of fluorescently-labeled actin

To fluorescently label the human β−actin used in Figures 3A and 3B, recombinant human globular β−actin (∼1.3 mg/mL) was polymerized in a buffer containing 2 mM MgCl_2_ and 100 mM KCl. The Alexa Fluor 488-C5-maleimide (Thermo Fisher Scientific, #A10254) dissolved in dimethyl sulfoxide (3 mM stock) was added to reach a 3.5 molar excess of dye to G-actin, and incubated for 1 hr at room temperature. The reaction was quenched with 10 mM DTT. F-actin was pelleted by using ultracentrifugation (Beckman TLA-55 rotor) at room temperature at 45000 rpm for 1 hr. The pellet was re-suspended and dialyzed against G-buffer to depolymerize actin. The free Alexa Fluor 488-maleimide was separated from labeled actin using Sephadex G-25 (GE Healthcare, PD MidiTrap G-25, #28-9180-07).

#### Actin polymerization assays

For human β-actin polymerization assay, 10 μL reaction mixture containing 2 μL G-buffer, 5 μL of 1 μM AlexaFluor 488-conjugated purified actin, 2 μL of 50 μM swinholide-A (or comparable volume of DMSO), 1 μL of 10x MKE were prepared.

For *S. pombe* actin polymerization assay, 10 μL reaction mixture containing 4.5 μL G-buffer, 2.5 μL of purified actin, 2 μL of 50 μM swinholide-A (or comparable volume of DMSO), 1 μL of 10x MKE were prepared.

The reaction mixtures were incubated at room temperature for at least 30 min. A further 0.5 μL of Rhodamine-phalloidin (Life Technologies, R415) was added to the reaction mixture containing *S. pombe* actin prior to imaging. One microliter of reaction mixture was sandwiched between a coverslip and a slide, and imaged using the spinning-disk confocal microscope at room temperature.

#### Analyses of F-actin severing by Swinholide A

Coverslips of two different sizes (40 × 22 mm and 20 × 20 mm; #1.5, Scientific Lab Supplies) were cleaned by washing in 2% (v/v) solution of Hellmanex III (Sigma, Z805939) for 30 min at 60°C, and then were rinsed three times with water and blow-dried with dry nitrogen gas. To make a flow chamber, two clean coverslips of different sizes were sandwiched together using double sided tapes (Nichiban 90 μm). The flow chambers were treated with mixtures of PLL-PEG (SuSoS PLL(20)-g[3.5]-PEG(2)) and PLL-PEG/PLL-PEG-Biotin (SuSoS PLL(20)-g[3.5]-PEG(2)/PEG(3.4)biotin20%) with final concentration of PEG-Biotin at 0.5% in HEPES Rehydration buffer (HRB, 20 mM HEPES [pH 7.1], 150 mM NaCl) for 1 hr at room temperature. Unbound PLL-PEG were washed twice with 20 μL of HRB. The flow chambers were perfused with 0.17 mg/mL Avidin (Sigma A3275; resuspended in HRB) and incubated for 30 min at room temperature.

Alexa Fluour-488-labeled human beta G-actin was mixed with unlabeled G-actin to a final labeling ratio of 10%, and then supplemented with Magnesium exchange buffer (100 mM Imidazole [pH 7.0], 50 mM MgCl_2_, 2 mM EGTA, 1 M KCl), and incubated on ice for 5 min. Actin polymerization was induced at room temperature for 15 min in polymerization buffer (20 μM labeled G-actin, 0.2 mM ATP, 0.5 mM DTT, 10 mM Imidazole [pH 7.0], 1 mM MgCl_2_, 1 mM EGTA, 50 mM KCl). Biotinylation of F-actin was achieved by incubating F-actin with Phalloidin-biotin (Invitrogen, Biotin-XX Phalloidin B-7474) to a final concentration of 200 nM for 10 min.

Prior to TIRF microscopy analyses, an aliquot of biotinylated F-actin was diluted with polymerization buffer to a final concentration of 50 nM, and perfused into the flow chambers. After incubation at room temperature for 10 min, the flow chambers were subjected to TIRF microscopy analyses. Excess F-actin were washed with one volume of HRB. For chemical compound treatments in Figure 3B and 1 μM Swinholide-A (Enzo BML-T125-0020) in HRB was used. DMSO was used in the equivalent volume as Swinholide-A as a control treatment.

#### Image analysis

Images were analyzed using Fiji. All image stacks except those in Figures 3A and 3B were projected along the Z axis (maximum intensity) for analysis and for representation. To measure the protein fluorescence intensities in Figure 1D, image stacks were projected along the Z axis (sum intensity) for quantification. The background of all microscopy images was subtracted in Fiji (Fiji/Process/Subtract Background). All time-lapse microscopy images were corrected for photo-bleaching in Fiji (Fiji/Image/Adjust/Bleach Correction).

To measure the R/S ratios, the longest diameter of the rings (indicated by Rlc1 or LifeAct fusion proteins) and the longest diameter of the spheroplasts were measured using the segmented line and measurements tools of Fiji. The shapes of the spheroplasts and the newly assembled rings were verified to be of a round shape by looking at the 3D projection (Fiji/Image/Stacks/3D project) of the spheroplasts and rings.

### Quantification and Statistical Analysis

Statistical significance was determined using Student’s t test in Figures 3D and 3F. Calculations of mean, standard deviation (s.d.), and statistical significances, were done using Prism 6.0 (GraphPad).

## Supplemental Information

Document S1. Figures S1 and S2

Movie S1. Wild-Type Spheroplasts Assemble an Equatorial Actin Ring, Related to Figure 1B Wild-type cells expressing LifeAct-EGFP (a proxy for actin filaments) and mCherry-Atb2 (tubulin) were imaged at 36°C.

Movie S2. *mid1*-18 *tea1*Δ Spheroplasts Assemble an Equatorial Actin Ring at the Restrictive Temperature, Related to Figure 2C Mutant cells expressing LifeAct-EGFP (a proxy for actin filaments) and mCherry-Atb2 (tubulin) were imaged at 36°C.

Movie S3. Severing of Actin Filaments by Swinholide-A, Related to Figure 3B Preassembled human β-actin filaments were treated with DMSO or swinholide-A and observed using TIRF microscopy at room temperature.

Movie S4. Actin Ring Assembly in the *mid1*-18 *tea1*Δ Spheroplasts Incubated with DMSO, Shown in Top Panel, and 10 μM Swinholide-A, Shown in Bottom Two Panels, at the Restrictive Temperature, Related to Figure 3C Mutant cells expressing LifeAct-EGFP (a proxy for actin filaments) and mCherry-Atb2 (tubulin) with different treatments were imaged at 36°C.

Movie S5. Actin Ring Assembly in the *mid1*-18 *tea1*Δ Spheroplasts along the Path of Least Curvature at the Restrictive Temperature, Related to Figure 4B Mutant cells expressing LifeAct-EGFP (a proxy for actin filaments) and mCherry-Atb2 (tubulin) were compressed and imaged at 36°C.

Document S2. Article plus Supplemental Information
