# Supplementary material for: Equatorial Assembly of the Cell-Division Actomyosin Ring in the Absence of Cytokinetic Spatial Cues
Source: Curr Biol. 2018 Mar 19;28(6):955–962.e3. doi: 10.1016/j.cub.2018.01.088 (PMC5863765; doi:10.1016/j.cub.2018.01.088)
Supplement: Document S1. Figures S1 and S2 [file mmc1.pdf]

Current Biology, Volume *28*

Supplemental Information

Equatorial Assembly of the Cell-Division Actomyosin Ring in the Absence of Cytokinetic Spatial Cues

Tzer Chyn Lim, Tomoyuki Hatano, Anton Kamnev, Mohan K. Balasubramanian, and Ting Gang Chew

Figure S1

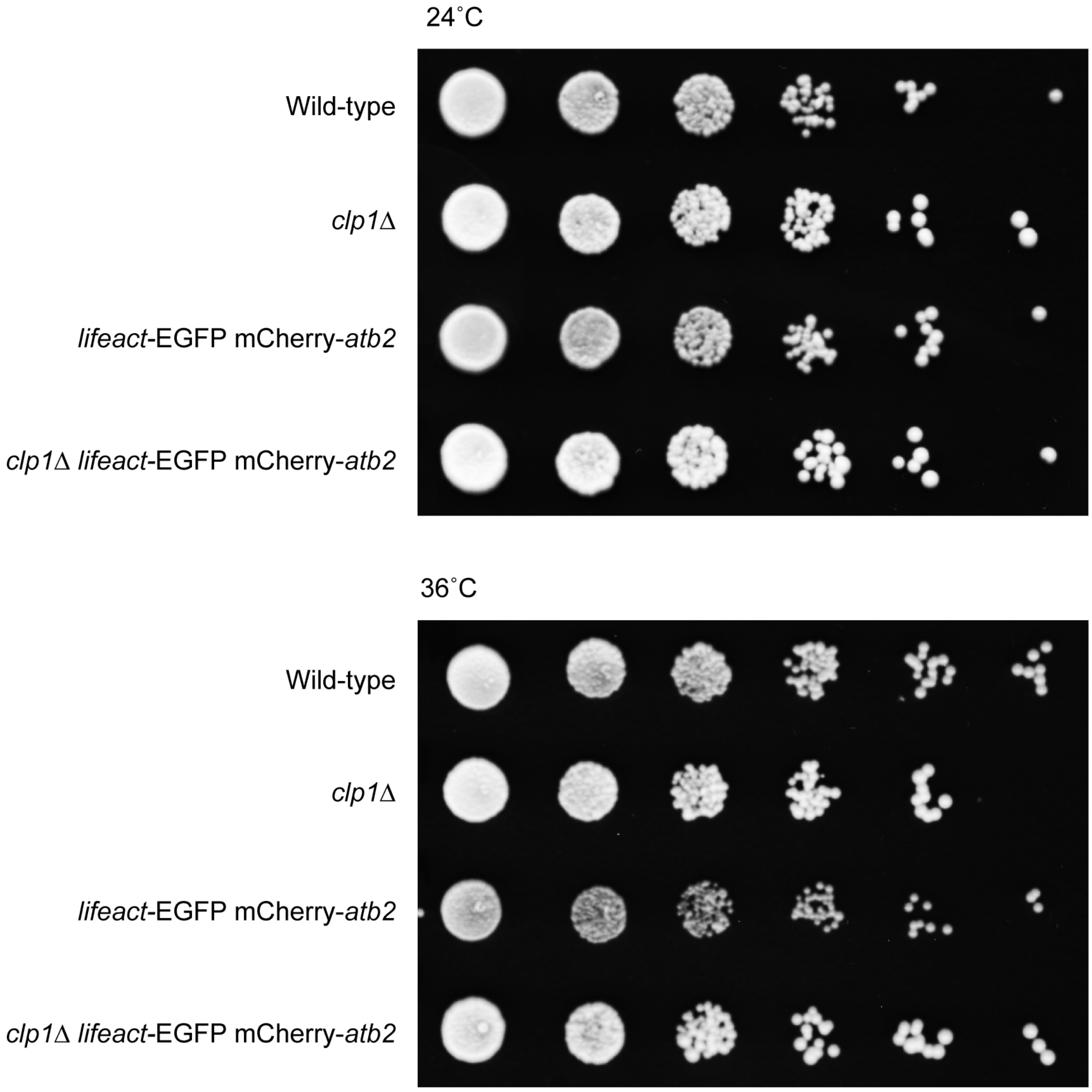
24˚C
Wild-type
*clp1*Δ
lifeact-EGFP mCherry-*atb2*
*clp1*Δ *lifeact*-EGFP mCherry-*atb2*
36˚C
Wild-type
*clp1*Δ
lifeact-EGFP mCherry-*atb2*
*clp1*Δ *lifeact*-EGFP mCherry-*atb2*

Figure S2

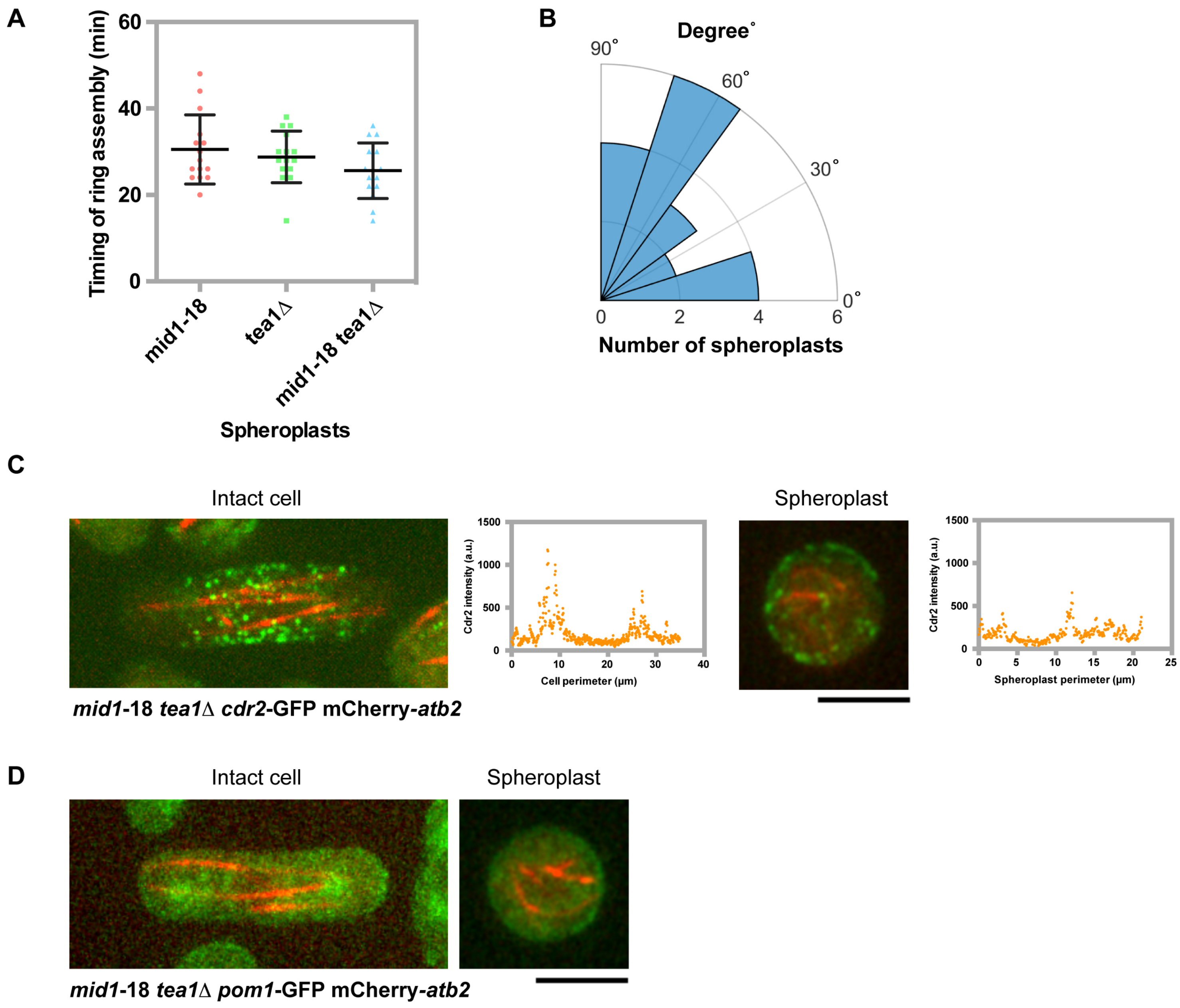

A
Timing of ring assembly (min)
0
20
40
60
mid1-18
tea1Δ
mid1-18 tea1Δ
Spheroplasts
B
Degree°
90°
60°
30°
0°
0
2
4
6
Number of spheroplasts
C
Intact cell
Cdr2 intensity (a.u.)
Cell perimeter (μm)
Spheroplast
Spheroplast perimeter (μm)
mid1-18 tea1Δ cdr2-GFP mCherry-atb2
D
Intact cell
Spheroplast
mid1-18 tea1Δ pom1-GFP mCherry-atb2

Figure S1. Analyses of the effects of LifeAct-EGFP expression in cells. Related to Figure 1.

Wild-type, *clp1*Δ, *lifeact-EGFP* mCherry-*atb2*, *clp1*Δ *lifeact-EGFP* mCherry-*atb2* cells were serially diluted, spotted on two YE agar plates, and incubated at 24°C and 36°C, respectively. The LifeAct-EGFP driven under the *S. pombe* actin promoter was used to label actin filaments. To test if expression of LifeAct-EGFP under the actin promoter caused any cytokinetic defects, LifeAct-EGFP was expressed in cells lacking Clp1 phosphatase. The clp1-null cells were previously shown to be sensitized for mild cytokinetic perturbation. There were no noticeable cytokinetic defects observed in clp1-null cells expressing LifeAct-EGFP.

Figure S2. Characterization of *mid1*-18 *tea1*Δ cells and spheroplasts. Related to Figure 2.

(A) Timing of ring assembly in *mid1*-18, *tea1*Δ, and *mid1*-18 *tea1*Δ spheroplasts.

(B) The inclination angles between the long axes of anaphase spindles and the plane of actomyosin rings in *mid1*-18 *tea1*Δ spheroplasts were measured and plotted (n = 20 spheroplasts).

(C) Localization of Cdr2-GFP in *mid1*-18 *tea1*Δ intact cells and spheroplasts. Fluorescence intensities of Cdr2-GFP were measured along the cell perimeter. The image at the focal plane was selected for the intensity measurement.

(D) Localization of Pom1-GFP in *mid1*-18 *tea1*Δ intact cells and spheroplasts.

Scale bar: 5 μm; error bars: s.d.
